# Glioblastoma Cell-Secreted Interleukin-8 Induces Brain Endothelial Cell Permeability via CXCR2

**DOI:** 10.1371/journal.pone.0045562

**Published:** 2012-09-20

**Authors:** Julie Dwyer, Jagoda K. Hebda, Armelle Le Guelte, Eva-Maria Galan-Moya, Sherri S. Smith, Sandy Azzi, Nicolas Bidere, Julie Gavard

**Affiliations:** 1 Cnrs, UMR8104, Paris, France; 2 Inserm, U1016, Paris, France; 3 Universite Paris Descartes, Sorbonne Paris Cite, Paris, France; 4 Inserm, U1014, Villejuif, France; Columbia University, United States of America

## Abstract

Glioblastoma constitutes the most aggressive and deadly of brain tumors. As yet, both conventional and molecular-based therapies have met with limited success in treatment of this cancer. Among other explanations, the heterogeneity of glioblastoma and the associated microenvironment contribute to its development, as well as resistance and recurrence in response to treatments. Increased vascularity suggests that tumor angiogenesis plays an important role in glioblastoma progression. However, the molecular crosstalk between endothelial and glioblastoma cells requires further investigation. To examine the effects of glioblastoma-derived signals on endothelial homeostasis, glioblastoma cell secretions were collected and used to treat brain endothelial cells. Here, we present evidence that the glioblastoma secretome provides pro-angiogenic signals sufficient to disrupt VE-cadherin-mediated cell-cell junctions and promote endothelial permeability in brain microvascular endothelial cells. An unbiased angiogenesis-specific antibody array screen identified the chemokine, interleukin-8, which was further demonstrated to function as a key factor involved in glioblastoma-induced permeability, mediated through its receptor CXCR2 on brain endothelia. This underappreciated interface between glioblastoma cells and associated endothelium may inspire the development of novel therapeutic strategies to induce tumor regression by preventing vascular permeability and inhibiting angiogenesis.

## Introduction

Glioblastoma is a highly aggressive and fatal brain tumor. Current treatments are largely palliative and mean patient survival post-diagnosis is approximately 12–15 months. Thus, improved treatment strategies are clearly needed. A major pathological feature of glioblastoma is extensive vascularization, the degree of which correlates with tumor stage [1]. Therefore, targeting mediators of tumor-angiogenesis has been proposed as a treatment for glioblastoma. Notably, expression of pro-angiogenic factors, such as vascular endothelial growth factor (VEGF), has been detected in the glioblastoma tumor microenvironment [2]. However, although promising, anti-VEGF therapies are not suitable for all patients, administration is expensive and severe adverse effects have been described [3]–[5]. Therefore alternate and/or combinatory therapies against other molecular pathways have to be designed.

Endothelial cells are required for tumor vascularization, as the ensuing delivery of oxygen and nutrients is critical for both expansion of malignant cells and maintenance of the cancer stem cell reservoir [3], [4], [6]. Of note, in glioblastoma, the endothelium is compromised and the leaky vasculature lends itself to increased interstitial fluid pressure and edema formation, while also limiting effective drug delivery [7]. Thus reversal of these characteristics by anti-angiogenic and/or normalization therapies holds substantial promise for improved treatment efficacy and possible cure. However, whilst VEGF plays a critical role in angiogenesis, especially in the regulation of endothelial permeability, the outcome of inhibiting its actions in clinical trials has been somewhat perplexing. There have been reports that anti-VEGF therapies, while reducing tumor blood supply, favor tumor cell invasion in glioblastoma [8]. In addition, despite a two-month increase in disease-free survival, use of the anti-VEGF monoclonal antibody, Bevacizumab, in combination with conventional therapies did not alter overall mortality [9]. Serious adverse effects on normal vasculature and fatal bleeding have been reported as well. Finally, there is a chance that up-regulation of compensatory mechanisms account for the lower than expected efficacy of anti-VEGF therapies. Thus, identification and investigation of other key regulators of permeability and angiogenesis in glioblastoma is required.

In glioblastoma, elevated concentration of the chemokine interleukin-8 (IL-8 or CXCL8) has been detected at the tumor resection margin. In comparison, IL-8 accumulation was found to be lower in the peritumoral region, thus indicating that this chemokine is associated with invasion and angiogenesis at the tumor border [10]. While initially characterized for its roles in immune regulation, it is now well accepted that IL-8 also functions as a potent pro-angiogenic factor, especially during tumorigenesis [11]. Indeed, increased levels of IL-8 have been detected in various tumors types, such as breast [12], melanoma [13] and glioblastoma [14], where its actions have been linked to tumor invasion, proliferation, survival and angiogenesis. With respect to its role in the latter, studies have demonstrated pro-proliferative, pro-survival and pro-angiogenic effects of recombinant IL-8 on endothelial cells [15], [16]. Endogenous IL-8, secreted by malignant colonic epithelial cells [17] and pancreatic cells [18], has also been shown to potentiate endothelial cell survival and other pro-angiogenic features such as tube formation. In addition, IL-8 provokes endothelial permeability [19], [20], which ultimately contributes to the angiogenic process, increased interstitial fluid pressure, edema formation, changes in blood flux and inefficient drug delivery [7]. However, while it is apparent that recombinant IL-8 imparts properties on macrovascular endothelium, the underlying dialogue taking place between brain tumor cells and local microvasculature requires further comprehension.

Herein, we have investigated the effects of the glioblastoma secretome using U87 glioblastoma cell line-derived conditioned media (CM) on the human brain microvascular endothelial cell line developed from a cancer-free patient, dubbed hCMEC/D3 [21]. We found that U87-CM induced both endothelial remodeling and permeability in association with increased extracellular signal-regulated kinase (ERK) signaling. Further analysis of the U87 secretome, by both ELISA and angiogenesis antibody array, revealed the presence of high levels of IL-8. Importantly, blockade of IL-8, by pharmacological inhibition of the IL-8 endothelial receptor CXCR2 or RNA interference-mediated silencing of the IL-8 messenger, significantly impaired the pro-permeability actions of U87-CM on brain microvascular endothelial cells *in vitro*. More in-depth exploration of the endothelial junctions uncovered a change in VE-cadherin stability upon stimulation of hCMEC/D3 with U87-CM, as demonstrated by dynamic reorganization of VE-cadherin localization away from cell junctions. Again this effect was arrested when IL-8/CXCR2 function was abrogated. Altogether, these findings support IL-8 as a key mediator of permeability in the glioblastoma microenvironment. More specifically, these data suggest that glioblastoma-derived IL-8 signals to brain microvascular endothelial cells *via* CXCR2, which promotes remodeling of VE-cadherin-mediated cell-cell junctions and increases permeability. Therapies targeting IL-8 could potentially decrease tumor-angiogenesis and reduce permeability to impair glioblastomagenesis and possibly optimize drug delivery.

## Materials and Methods

### Cell Culture, Preparation of Conditioned Media and siRNA Transfection

U87 MG, U138 MG, U251 MG and LN229 cells were obtained from the ATCC and maintained in MEM supplemented with 10% FBS in the presence of 1% non-essential amino acid, 1% glutaMAX, 1% sodium pyruvate and 1% penicillin/streptomycin (all from Invitrogen). Human cerebral microvascular endothelial cells, hCMEC/D3, were kindly donated by Dr. PO Couraud [21]. They were cultured in EBM2 (Lonza) supplemented with 5% Gold serum (PAA Lab), 1.4 µM hydrocortisone (Sigma), 5 µg/ml ascorbic acid (Sigma), 1 ng/ml basic Fibroblast Growth Factor (bFGF, Sigma), 1% chemically defined lipid concentrate (Invitrogen), 10 mM HEPES (Invitrogen), and 1% penicillin/streptomycin (Invitrogen).

For starvation, cells were washed thrice with PBS and incubated at 37°C/5% CO_2_-in-air overnight in MEM or EBM2 serum-free media for glioblastoma and endothelial cells, respectively. For conditioned media (CM), the following day media were decanted and cleared by centrifugation at 1000 rpm, 5 min, followed by filtration through a 22 µm filter. CM were then used immediately or stored at −20°C until use.

For IL-8 gene silencing, three commercially available siRNA duplexes against IL-8 were purchased (Stealth siRNA, Invitrogen). The duplex sequences were as follows: siRNA#1 (CCAAGGAGUGCUAAAGAACUUAGAU), siRNA #2 (GGUGCAGAGGGUUGUGGAGAA GUUU), and siRNA #3 (GCUCUCUUGGCAGCCUUCCUGAUUU). For CXCR1 gene silencing the duplex sequences used were as follows: siRNA #1 (GUUCUUGGCACGUCAUCGU), siRNA #2 (CACUUCUUCGUCUGUCAAU) (Sigma) and for CXCR2 gene silencing, they were: siRNA #1 (CAAACUGGCGGAUGCUGUUACGGAU) and siRNA #2 (AAGGACCGUCUACUCAUCCAAUGUU). Non-silencing (Low GC Duplex) was used as control (Invitrogen). U87 cells were transfected with 50 nM siRNA using RNAi max (Invitrogen) as per the manufacturer’s instructions. Seventy-two hours after transfection, cells were starved overnight as described above, and both CM and RNA were collected the following morning.

### Reagents and Antibodies

Recombinant human IL-8 and VEGF was purchased from PeproTech. SB225002 (CXCR2 inhibitor) was from Calbiochem and Ki8751 (VEGFR2 inhibitor) was from Sigma. The following antibodies were used: phospho-ERK1/2, phospho-IκBα, IκBα, phospho-p38, phospho-JNK and c-Jun (Cell Signaling Technologies), ERK2, VE-cadherin, p65, JNK, CXCR1, CXCR2, mouse pre-immune and α-tubulin (Santa Cruz), IL-8 (Peprotech) and phospho-S665 VE-cadherin [22].

### RNA Extraction and RT-PCR

RNA was extracted using the Qiagen RNeasy Mini Kit as per the manufacturer’s directions. Equal amounts of RNA were reverse transcribed using the Superscript III RT kit (Invitrogen) and the resulting cDNA was used to amplify IL-8, CXCR1 and CXCR2 mRNA by PCR using gene specific primer sets (IL-8∶5′-TAGCAAAATTGAGGCCAAGG-3′ and 3′-AAACCAAGGCACAGTGGAAC-5′; CXCR1∶5′-TTGCACAGACTGGCATTAGC-3′ and 3′-CTGTGACTTAGCACCACCAC-5′; CXCR2∶5′-ACAGCTACTTGGGAGGCTGA-3′ and 3′- TGCAGTGGTCACACCATTTT-5′) in the presence of Taq DNA polymerase (Invitrogen). Human beta-actin was also amplified as a control for input (β-actin: 5′-AGCACTGTGTTGGCGTACAG-3′ and 3′-GGACTTCGAGCAAGAGATGG-5′). PCR products were separated by electrophoresis on SYBR green-containing agarose gels (Invitrogen) and DNA was visualized by UV illumination (Appligene).

### Western Blotting

Western blotting was conducted using standard laboratory protocols [23]. Briefly, equal amounts of protein were separated on 4–20% polyacrylamide Nupage gels (Invitrogen) and transferred to PVDF membranes (Millipore). Membranes were incubated in 5% skim milk/TBST blocking solution for approximately 1 h followed by incubation with specific antibodies diluted in 2.5% milk/TBST blocking solution, on a rocking platform overnight at 4°C. Membranes were washed thrice in TBST, then incubated with the appropriate Alexa680-conjugated secondary antibody (Invitrogen) and scanned using the Odyssey Infra-Red Imaging System (Li-Cor BioSciences).

### Permeability Assay

*In vitro* permeability assays were conducted as described previously [22]. Briefly, 100000 hCMEC/D3s were seeded on 3 µm pore-size collagen-coated PTFE membranes (Costar, Corning). Permeability was then monitored by FITC-dextran 40 kDa (1 mg/ml, Invitrogen) passage five days later on a fluorescence plate reader (Fusion, Packard).

### Tubulogenesis Assay

Equal volumes of Matrigel (BD Biosciences) were added to wells of a 96-well plate and polymerized by incubation at 37°C for 30 min. Meanwhile, hCMEC/D3s were pre-incubated with CM, also at 37°C for 30 min. For experiments with SB225002 (CXCR2 inhibitor), a final concentration of 200 nM was used. Following 30 min pre-incubation, hCMEC/D3 suspensions were layered over Matrigel. Images were captured at regular time intervals over a 48 h period (Motic AE21 microscope). Tube formation and number of branching points were quantified using ImageJ software (NIH, USA).

### Measuring IL-8 Secretion

The RayBiotech angiogenesis antibody array (RayBiotech, TEBU) was used to examine the angiogenic secretome of U87 glioblastoma cells as per the manufacturer’s instructions. In addition, the concentration of IL-8 in various CM was quantified using the human IL-8 ELISA development kit (Peprotech) as per the manufacturer’s directions.

### Luciferase Reporter Assay

Firefly luciferase constructs downstream of promoters for Nuclear Factor κB (NF-κB) or activator protein 1 (AP-1) were co-transfected with renilla luciferase pRL-TK plasmid (Promega). Luciferase activity was analyzed using the Dual-Luciferase Kit (Promega), where firefly fluorescence units were normalized to renilla luciferase fluorescence units (BMG microplate reader).

### Immunofluorescence and Confocal Microscopy

Staining was performed on cells grown on collagen-coated coverslips and fixed with 4% paraformaldehyde. Briefly, cells were permeabilized with 0.5% Triton-PBS, blocked with 3% BSA-PBS and then incubated with primary antibodies diluted one hundred-fold in blocking solution. After several washes with PBS, cells were incubated with the appropriate Alexa488-conjugated secondary antibody diluted four hundred-fold (Invitrogen). Following more washes, cells were counterstained with 4′, 6′-diamidino-2-phenylindol (DAPI, Invitrogen, 1/10000 dilution) and mounted on glass slides with Fluoromount (Sigma). Confocal acquisitions were performed on a TCS/SP6 Leica confocal microscope (Institut Andre Lwoff, Villejuif, France). Three-dimensional pictures were reconstructed from 0.5 µm thick z-stacks (Imaris software, Cochin Imaging Facility, Paris, France).

### Flow Cytometry

siRNA-mediated knockdown of CXCR1 and CXCR2 in hCMEC/D3 was monitored by flow cytometry. Briefly, cells were suspended in DMEM and stained with indicated antibodies. Cells were subsequently washed 3 times with PBS and incubated for 30 min with AlexaFluor488-conjugated donkey anti-mouse antibody (Invitrogen). Cells were then fixed with paraformaldehyde (1% in PBS, 20 min, RT) and analyzed by flow cytometry. Fluorescence data was acquired for 10000 cells on a FACScalibur flow cytometer (BD Biosciences) and analyzed using CellQuest software (BD Biosciences). Non-transfected cells (basal) and cells stained with IgG alone served as controls.

### MTT (1-(4,5-dimethylthizol-2-yl)-3,5-diphenylformazan, Thiazolyl Blue Formazan) Assay

The survival of hCMEC/D3 was assessed by MTT as described in [24]. Briefly, hCMEC/D3 were seeded in 96-well flat bottom plates and allowed to adhere. Twenty-fours later, media were aspirated and cells were placed in 200 µl of regular media (Pos) or U87-CM (CM). The following day, the MTT assay was performed as per the manufacturer’s instructions (Sigma).

## Results

### The U87 Glioblastoma Cell Secretome Regulates Brain Endothelial Cell Permeability

**Figure 1:**
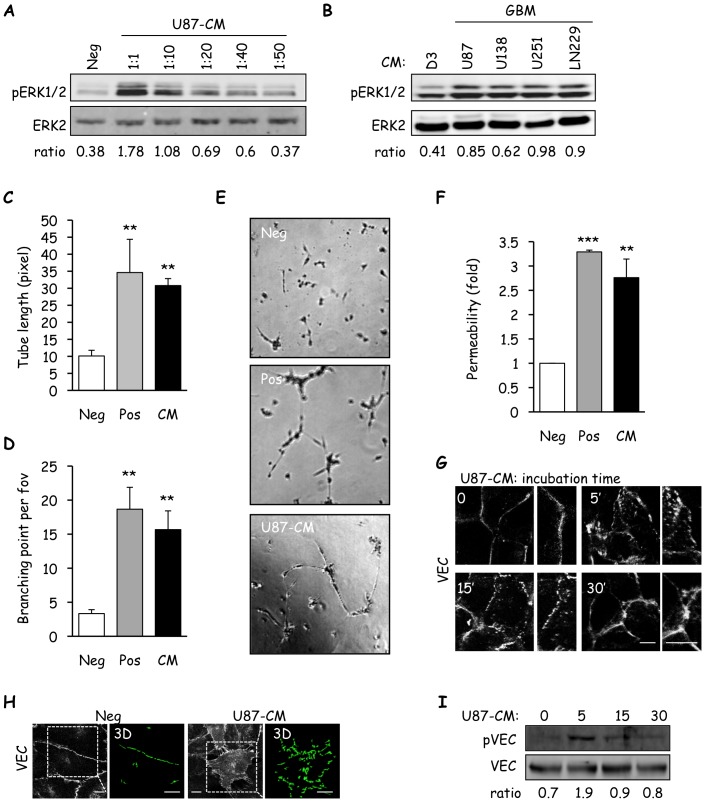
Glioblastoma cell-secreted factors induce loss of brain endothelial monolayer integrity. A) Starved human cerebral microvascular endothelial cells (hCMEC/D3) were incubated for 5 min with serum-free media (Neg) or U87-conditioned media (CM), diluted as indicated. Protein lysates were examined by western-blot for phosphorylated (p) ERK1/2 and ERK2. Ratios between pERK1/2 and ERK2 intensities are indicated below scans. B) Similarly, ERK1/2 phosphorylation was assessed in hCMEC/D3 stimulated with CM derived from glioblastoma cell lines (GBM, U87, U138, U251 and LN229), where hCMEC/D3-CM (D3) served as control. C–E) *In vitro* tubulogenesis of hCMEC/D3 was scored after incubation in matrigel with serum free-media (Neg), 50 ng/ml each VEGF/bFGF (Pos) or U87-CM (U87). After 8 h of incubation, cells were photographed and both tube length (C) and number of branch points per field of view (FOV) (D) were quantified. Representative images are shown. F) Using similar conditions, permeability to FITC-dextran was measured after 1 h incubation on hCMEC/D3 monolayers. Graph shows the mean fold-increase normalized to Neg conditions. G–H) Localization of VE-cadherin in response to stimulation with U87-CM at indicated time points was analyzed by confocal microscopy (G), and three-dimensional (3D) images were reconstructed using z-stacks (H). Scale bars: 5 µm (G) and 20 µm (H). I) VE-cadherin phosphorylation on S665 (pVEC) was analyzed by western-blot in hCMEC/D3 stimulated with U87-CM for the indicated times. Total VEC served as a loading control. One out of three independent experiments is shown. Ratios between pVEC and VEC intensities are indicated below scans. T test on 3 independent experiments: *p<0.05; **p<0.01; ***p<0.001.

To investigate the influence of glioblastoma secreted factors on human brain endothelial cells, the effects of conditioned media (CM) derived from U87 glioblastoma cells on several characteristics of brain microvascular endothelial cells (hCMEC/D3) were tested. First, U87-CM was able to induce ERK1/2 activation in quiescent monolayers of hCMEC/D3 and this activity remained intact up to a dilution of 1 in 50 (Fig. 1A). This effect was not restricted to U87 cells, since CM from additional glioblastoma cell lines also readily elicited ERK activation (Fig. 1B). In sharp contrast, CM derived from hCMEC/D3 did not greatly increase ERK phosphorylation when compared to the effects of glioblastoma cell line-derived CM (Fig. 1B), suggesting that glioblastoma-secreted factors specifically activate ERK. We next studied the impact of U87-CM on hCMEC/D3 tubulogenesis *in vitro*. CM from U87 cells induced more than twice the amount of both tubule formation and branching when compared to serum-free media (Neg) (Fig. 1C–E). Interestingly, U87-CM-driven tubulogenesis was almost equal to VEGF/bFGF stimulation (Pos) (Fig. 1C–E). Induction of tubulogenesis was also accompanied by a significant increase of hCMEC/D3 permeability, as evidenced by a 2.5-fold augmentation of FITC-dextran passage (Fig. 1F). To more comprehensively investigate the effects of U87-CM on endothelial integrity, the cell-cell junctions of hCMEC/D3 were examined. As expected, VE-cadherin accumulated essentially at endothelial junctions under basal conditions (0 min, Fig. 1G). In contrast, hCMEC/D3 exposed to U87-CM for 5–15 min, exhibited rapid redistribution of VE-cadherin into punctuated dots at cell-cell junctions and into the cytosol. This was even more striking when three-dimensional images were reconstructed (Fig. 1H). However, by 30 min VE-cadherin localization was restored to close to that found in the basal state (Fig. 1G). Notably, increase in VE-cadherin serine-phosphorylation – a signature of VE-cadherin internalization [22], [25] – aligned with its disorganized pattern in response to U87-CM (Fig. 1I). Taken together, these data suggest that U87-secreted factors impart a phenotype on hCMEC/D3 that recapitulates angiogenic features, including ERK activation, tubulogenesis, increased permeability and dynamic remodeling of VE-cadherin-mediated cell-cell junctions.

### Glioblastoma Cells Secrete High Levels of IL-8

**Figure 2:**
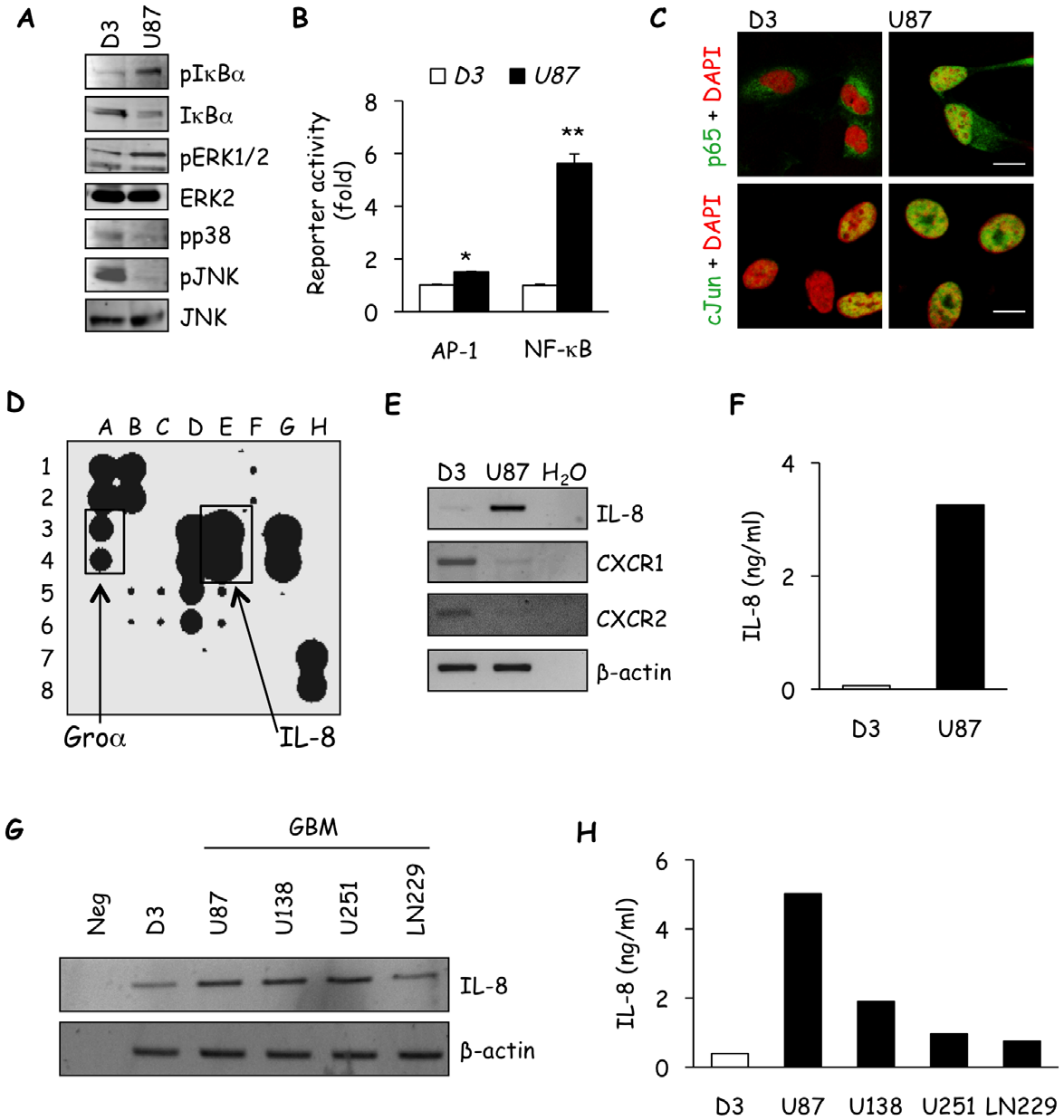
The glioblastoma cell secretome contains high levels of IL-8. A) The status of the NF-κB (pIκBα and IκBα) and MAPK (pERK1/2, ERK2, pp38, pJNK, and JNK) signaling pathways were assessed in lysates from serum-starved hCMEC/D3 (D3) and U87 by western-blot. B) Promoter activity of both AP-1 and NF-κB in D3 and U87 were determined by luciferase reporter assays. C) Immunofluorescent staining for NF-κB p65 (green) and c-Jun (green) was performed as readout of NF-κB and AP-1 signaling activity, respectively, in serum-starved D3 and U87. DAPI (red) was used to stain nuclei, scale bars: 10 µm. D) The angiogenic secretome profile was determined in U87 conditioned medium (CM) using antibody array, IL-8 and its closely related family member, Gro-α, are indicated. E) The mRNA levels of IL-8, CXCR1 and CXCR2 cells were determined in U87 and D3 by RT-PCR. β-actin was used as a control for loading and PCR efficacy. F) IL-8 secretion in conditioned media from U87 and D3 was examined through ELISA. G) Expression of the IL-8 transcript was examined in D3, U87 and additional glioblastoma cell lines (GBM, U138, U251 and LN229) by RT-PCR. H) Similarly, IL-8 secretion was measured in these cell lines by ELISA. One out of three independent experiments is shown. T test on 3 independent experiments: *p<0.05; **p<0.01.

Because cytokine secretion is essentially controlled by the concerted actions of NF-κB and AP-1 signaling [26], [27]; the statuses of these pathways were examined in both U87 and hCMEC/D3. We first monitored IκBα, which undergoes phosphorylation and degradation to unleash NF-κB translocation into the nucleus. We found that U87 exhibited greater phosphorylation and degradation of IκBα than hCMEC/D3, suggesting that the NF-κB pathway is more activated in U87 cells (Fig. 2A). In correspondence with this data, the NF-κB promoter was five times more active in U87 than hCMEC/D3, and NF-κB p65 was more accumulated within U87 nuclei than hCMEC/D3 (Fig. 2B–C). Although modest, the activation of the AP-1 promoter was also found to be higher in U87 than in hCMEC/D3, and more c-Jun was present in U87 nuclei (Fig. 2B–C). Thus, these data point towards increased NF-κB and AP-1 activity as key promoters of cytokine gene transcription in U87. MAPK activation was also examined in detail. We found that ERK activity was higher in U87. By contrast, phosphorylations of p38 and JNK were lower when compared to D3 (Fig. 2A). This suggests that AP-1 activity mainly relies on ERK1/2 in U87.

To illuminate the angiogenic factors secreted by U87, CM was analyzed using an unbiased angiogenesis-specific antibody array targeting 20 candidates. Among the ten pro-angiogenic factors readily detected, the most elevated was the chemokine IL-8 (Fig. 2D and Table 1). In fact, IL-8 levels exceeded that of the positive controls. Other factors that were also high, included growth regulated oncogene-alpha (Gro-α), which shares receptors with IL-8, Monocyte Chemotactic Protein (MCP-1, also known as CCL2), interleukin-6 (IL-6) and Tissue Inhibitor of MetalloProteases-1 (TIMP). Lower levels of Epidermal Growth Factor (EGF), Regulated upon Activation Normal T-cell Expressed and Secreted (RANTES or CCL5), TIMP-2, Vascular Endothelial Growth Factor A (VEGF-A), and Transforming Growth Factor beta-1 (TGFβ-1) were also measured (Fig. 2D and Table 1). As increasing evidence supports IL-8 as a major pro-angiogenic factor, the role of this chemokine in crosstalk between glioblastoma cells and brain endothelial cells was examined. First, RT-PCR analysis revealed higher IL-8 mRNA in U87 than in hCMEC/D3, while an inverse pattern was observed in the case of the IL-8 receptors, CXCR1 and CXCR2 (Fig. 2E). Furthermore, ELISA confirmed that U87 glioblastoma cells secrete high levels of IL-8 (Fig. 2F). Such results were extended to multiple glioblastoma cell lines, where three additional glioblastoma cell lines (U138, U251 and LN229) were found to express IL-8 mRNA and secrete the protein to various extents (Fig. 2G–H). Of note, LN229, which had the lowest IL-8 mRNA expression, also secreted less IL-8. The difference in IL-8 mRNA expression and secretion, among the glioblastoma CM, is likely the result of tumor heterogeneity. Thus, elevated IL-8 emerges as a potential feature of glioblastoma cell lines, while by contrast, hCMEC/D3 transcribe and secrete relatively low levels under normoxic conditions. This dichotomy in the expression of IL-8 and its receptor CXCR2 might explain how glioblastoma cells regulate brain endothelium properties.

**Table 1:** Glioblastoma cell-secreted angiogenic factors.

Reference	Protein	Intensity (AU)	Reference	Protein	Intensity (AU)
A1–A2	POS	671	E1–E2	Angiogenin	0
**A3–A4**	**Gro-α**	**439**	**E3–E4**	**IL-8**	**1559**
A5–A6	PIGF	0	**E5–E6**	**TIMP-2**	**87**
A7–A8	BLANK	0	E7–E8	BLANK	0
B1–B2	POS	726	**F1–F2**	**EGF**	**60**
B3–B4	IFNγ	0	F3–F4	LEPTIN	0
**B5–B6**	**RANTES**	**52**	F5–F6	Thrombopoietin	0
B7–B8	BLANK	0	F7–F8	BLANK	0
C1–C2	NEG	0	G1–G2	ENA-78	0
C3–C4	IGF-1	0	**G3–G4**	**MCP-1**	**1106**
**C5–C6**	**TGFβ-1**	**52**	**G5–G6**	**VEGF-A**	**17**
C7–C8	BLANK	0	G7–G8	NEG	0
D1–D2	NEG	0	H1–H2	bFGF	0
**D3–D4**	**IL-6**	**854**	H3–H4	PDGF-BB	0
**D5–D6**	**TIMP-1**	**518**	H5–H6	VEGF-D	0
D7–D8	BLANK	0	H7–H8	POS	771

A representative scan of the angiogenesis array film was analyzed using ImageJ analysis software. Reference refers to the position on the array as reported on figure 2D. Density represents mean arbitrary values corresponding to pixel intensity for each spot.

POS: positive control; Gro-α: growth-regulated oncogene; PlGF: Placenta growth factor; IFN: interferon; RANTES: regulated upon activation, normal T-cell expressed, and secreted; NEG: negative control; IGF: insulin-like growth factor; TGF: transforming growth factor; IL: interleukin; TIMP: tissue inhibitor of metalloproteinases; EGF: epidermal growth factor; ENA-78: epithelial neutrophil-activating peptide 78; MCP-1: monocyte chemotactic protein-1; VEGF: vascular endothelial growth factor; bFGF: basic fibroblast growth factor; PDGF: platelet-derived growth factor.

### Expression of IL-8, but not CXCR2, is Elevated in Human Glioblastoma

**Figure 3:**
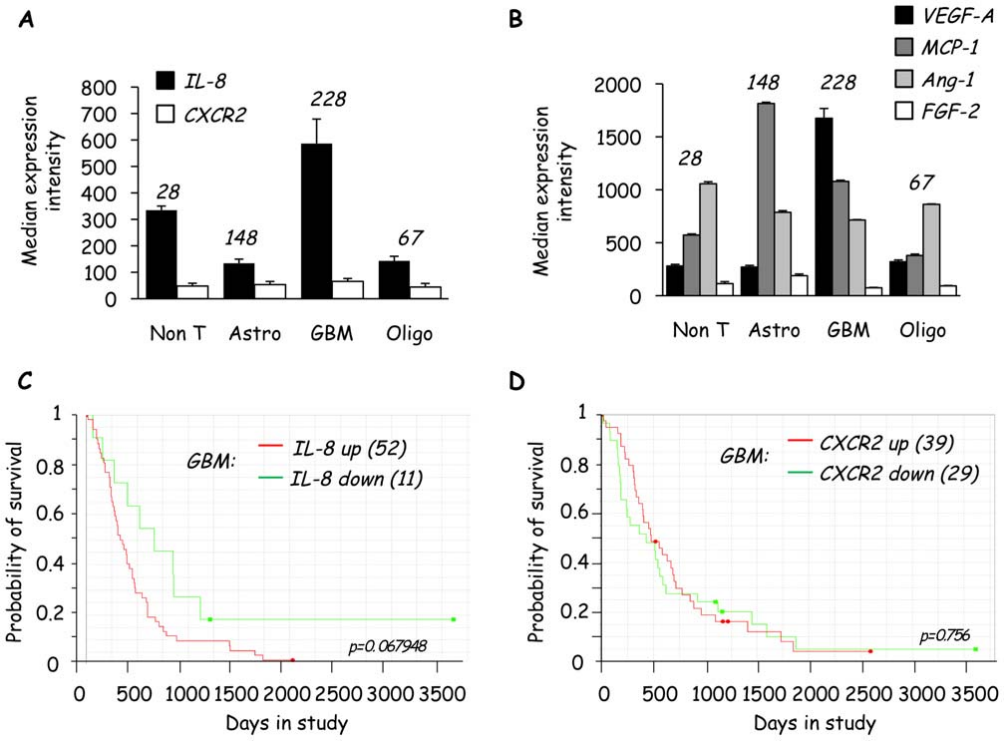
Expression of IL-8, but not CXCR2, is elevated in human glioblastoma. A–D) Retrospective gene array analysis was performed using the REMBRANDT (Repository of Molecular Brain Neoplasia Data) collection from National Cancer Institute (NCI) and the National Institute of Neurological Disorders and Stroke (NINDS). Median expression intensities are reported as a graph for IL-8 and CXCR2 genes in (A) and for VEGF-A, MCP-1 (Monocyte Chemotactic Protein), Ang-1 (angiopoeitin) and FGF-2 (fibroblast growth factor) genes in (B), on Non T: non tumor tissue; Astro: astrocytoma; GBM: glioblastoma multiforme; Oligo: oligodendroglioma. C–D) Probability of survival is shown for GBM divided into groups with either high (3-fold increase) or low (3-fold decrease) gene expression of IL-8 (C) and CXCR2 (D). p values (p) are provided. N indicates sample number in each category. All data were obtained using the REMBRANDT database accessed on June 11^th^ 2012.

To further explore the relationship between IL-8 and glioblastoma, the Rembrandt database was used to search existing human gene array library concerning expression of IL-8 and CXCR2 in glioblastoma and other brain tumor types. Interestingly, IL-8 was particularly heightened in glioblastoma in comparison to non-tumor brain tissues or other brain tumor types, such as astrocytoma and oligodendroglioma. In contrast, expression of CXCR2 remained uniform across the different brain biological samples (Fig. 3A). This trend suggests that IL-8 might be the rate-limiting factor for IL-8/CXCR2 activity. The median expression intensity of other key angiogenic factors, VEGF-A, Ang-1 (Angiopoietin 1) and FGF-2 (basic fibroblast growth factor), were also determined using pooled gene array datasets on Rembrandt (Fig. 3B). VEGF-A messenger was elevated in glioblastoma when compared with other tumor types and non-tumors. However, this retrospective analysis did not unveil obvious difference in the median expression intensity of Ang-1 and FGF-2. MCP-1 was also investigated, as it was readily detected in our antibody array and its expression has been linked to tumor progression [28]–[30]. Indeed, MCP-1 appears to be higher in astrocytoma and glioblastoma, but not oligodendroglioma, when compared with non-tumor tissues (Fig. 3B). These data reiterate the close relationship between angiogenesis and tumorigenesis and support their involvement in brain tumors. Next, to determine whether IL-8 levels within the tumor mass are related to patient survival, this parameter was analyzed in glioblastoma patients with either high or low IL-8, where a 3-fold increase or decrease was set as a threshold for up- and down-regulated genes, respectively. It was found that elevated IL-8 correlates with a reduced probability of survival (Fig. 3C), suggesting that increased IL-8 might reflect a more aggressive glioblastoma subtype. Interestingly, IL-8 expression level was higher in approximately 80% of patients (52/63) patients with glioblastoma (Fig. 3C). Again, variation in CXCR2 expression levels did not influence the probability of patient survival (Fig. 3D). Therefore, IL-8, but not CXCR2 expression, might contribute to glioblastoma aggressiveness and high vascularity in human patients.

### IL-8 Mimics the Effects of the Glioblastoma Secretome on Brain Endothelial Cells

**Figure 4:**
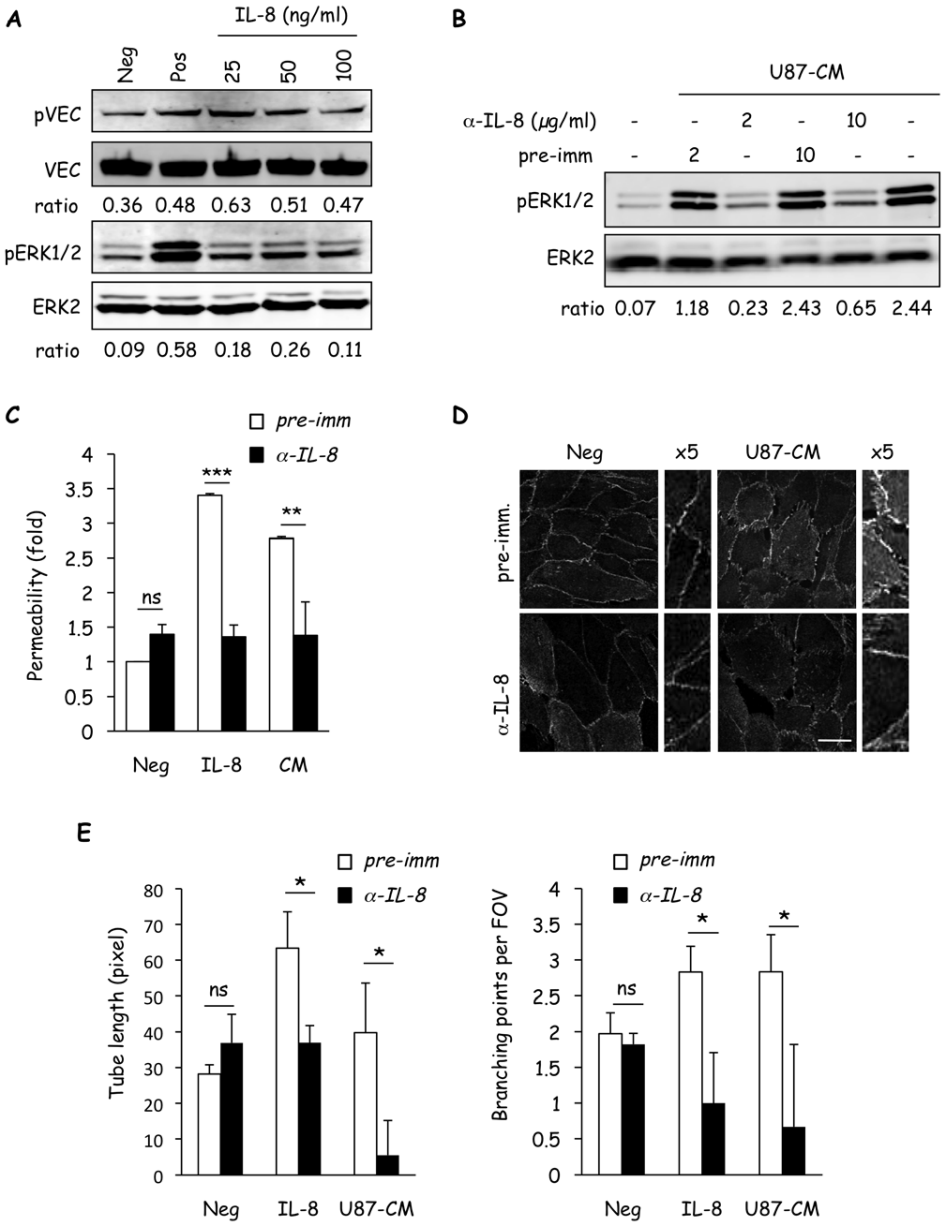
IL-8 mimics the effects of the glioblastoma secretome on brain endothelial cells. A) The status of VE-cadherin (pVEC and VEC) and ERK (pERK1/2 and ERK2) were examined by western-blot in hCMEC/D3 stimulated with recombinant IL-8 (25–100 ng/ml, 5 min). Starvation media and serum-containing media were used for negative (Neg) and positive (Pos) controls, respectively. Ratios between pVEC and VEC and pERK1/2 and ERK2 intensities are indicated below scans. B) Two doses of anti-IL-8 blocking antibody (a-IL-8) and of pre-immune serum (pre-imm.) were tested for their effect on U87-CM-induced activation of ERK (pERK1/2) by western-blot. Total ERK2 was used as a control for loading. Ratios between pERK1/2 and ERK2 intensities are indicated below scans. C–E) Similarly, the effects of IL-8 blocking antibody (2 µg/ml) were examined on recombinant IL-8- or U87-CM-induced permeability (C), VE-cadherin localization (D) or tubulogenesis (E).

To further investigate the role of IL-8 in U87-CM-induced effects on hCMEC/D3; recombinant IL-8 protein and IL-8-specific antibody were employed. First, recombinant IL-8 peptide induced ERK activity and serine phosphorylation of VE-cadherin. A dose of 25 ng/ml IL-8 was sufficient to induce phosphorylation of both ERK and VE-cadherin within 5 minutes (Fig. 4A). The optimized dose of 50 ng/ml IL-8 was used, as higher doses were either inhibitory or had no additional effects. Next, we tested whether blocking glioma cell-secreted IL-8 bioactivity and availability with specific antibody could interfere with the effects of U87-CM on ERK activity in hCMEC/D3. As before, U87-CM induced ERK phosphorylation, however, pre-incubation of U87-CM with IL-8 antibody readily impaired the downstream activation (Fig. 4B). Similarly, recombinant IL-8 mirrored the effects of U87-CM by increasing permeability, redistributing VE-cadherin, and enhancing tubulogenesis of hCMEC/D3 (Fig. 4C–E). Importantly, these phenotypes could be prevented or reduced with IL-8-specific antibody pre-incubation (Fig. 4C–E).

### U87 Glioblastoma-secreted IL-8 Modulates Brain Endothelial Cell Properties

**Figure 5:**
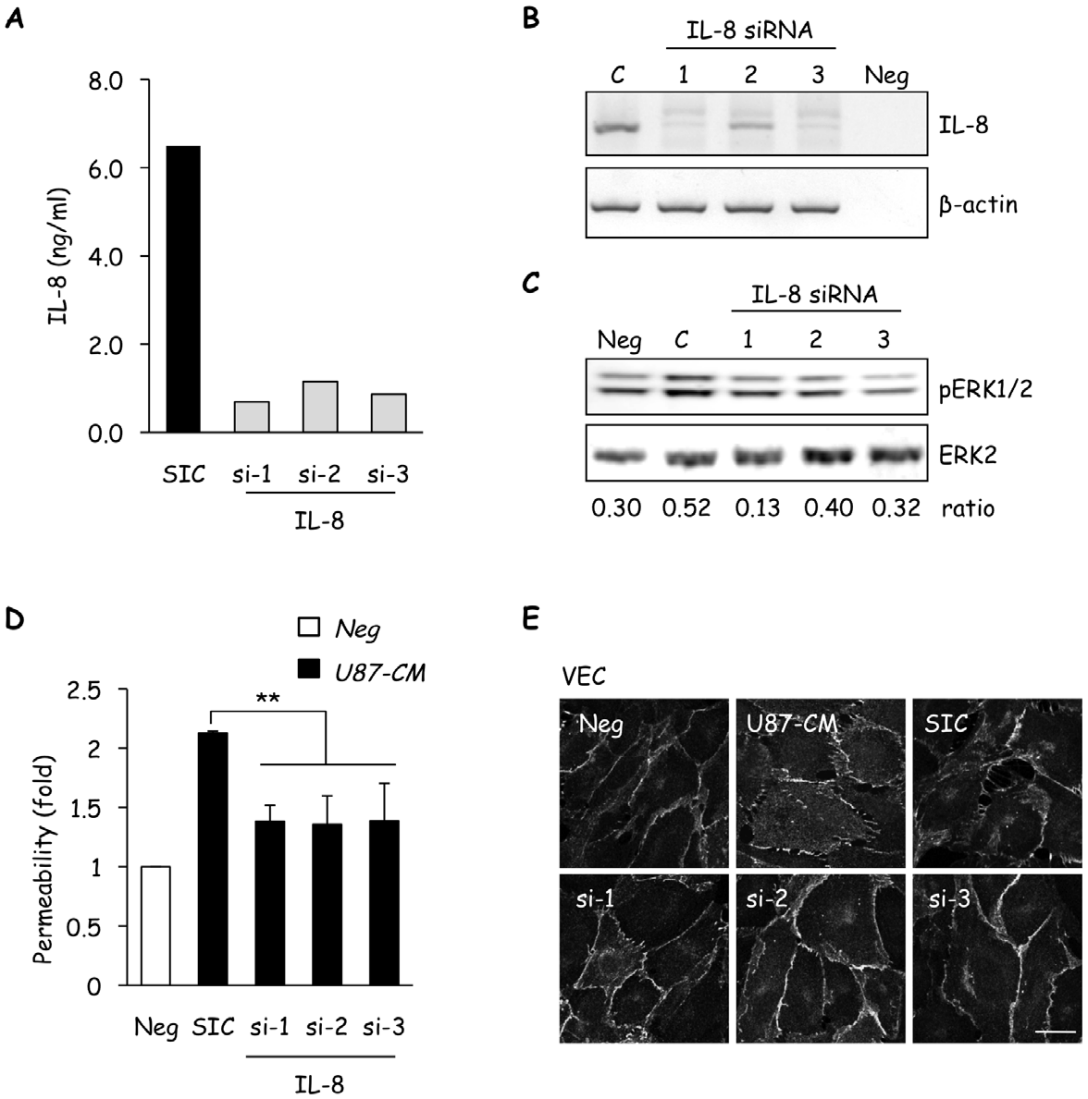
Glioblastoma cell-secreted IL-8 modulates brain endothelial cell properties. A–B) IL-8 secretion was decreased in U87 using three independent IL-8-targeting siRNA (si-1, si-2, and si-3) with the non-silencing sequence (SIC) serving as control. IL-8 siRNA efficiency was confirmed by ELISA (A) and RT-PCR (B). C–E) Three day-old starved human cerebral microvascular endothelial cells (hCMEC/D3) were stimulated with U87 conditioned medium (CM, 1/20 diluted) collected 72 h after transfection with IL-8 siRNAs, and analyzed for ERK activation (pERK1/2) by western-blot (C), permeability to FITC-dextran (D), and VE-cadherin (VEC) immunolocalization (E). Ratios between pERK1/2 and ERK2 intensities are indicated below scans. Scale bar: 10 µm. One out of three independent experiments is shown. T test on 3 independent experiments: **p<0.01.

To unambiguously examine the role of secreted IL-8 in U87-CM-induced plasticity of hCMEC/D3, IL-8 was silenced by RNA interference with three individual siRNA sequences. ELISA and RT-PCR experiments were performed to validate the efficient knockdown of IL-8 (Fig. 5A–B). When exposed to CM-derived from IL-8-silenced U87 cells, ERK phosphorylation in hCMEC/D3 was dampened (Fig. 5C). Moreover, knockdown of IL-8 also reduced U87-CM-induced permeability of hCMEC/D3, and prevented VE-cadherin redistribution (Fig. 5D–E). Hence, our data suggest that IL-8 is critical for U87-CM-driven disruption of VE-cadherin-regulated endothelial junctions, which likely mediates increased permeability.

### CXCR2, but not CXCR1, is Required for U87-CM-induced Permeability

**Figure 6:**
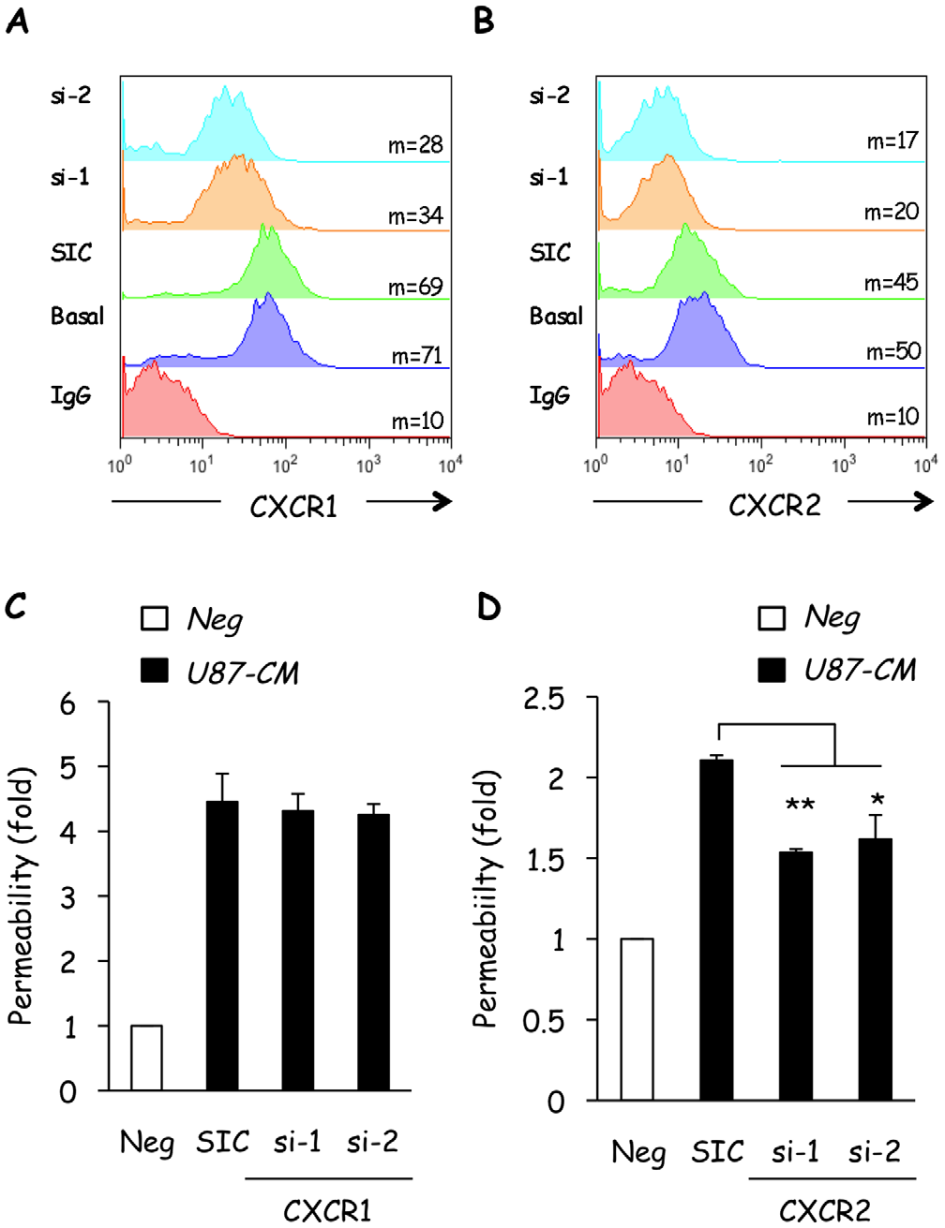
CXCR2, but not CXCR1, is required for U87-CM-induced permeability. A–B) Human cerebral microvascular endothelial cells (hCMEC/D3) were transfected with CXCR1- and CXCR2-targeting duplexes (si), and non-targeting control siRNA (SIC). Flow cytometric analysis of CXCR1 and CXCR2 expression was performed 72 h later. Non-transfected cells (basal) and IgG alone served as controls. Mean fluorescence intensity is indicated (m). Offset histograms were prepared for comparison of all conditions. C–D) Similarly transfected cells were seeded on collagen-coated permeability inserts and permeability to FITC-dextran was measured 72 h later. Graph shows the mean fold-increase normalized to serum-starved cells (Neg). T test on 3 independent experiments: **p<0.01; *p<0.05.

We next aimed to pinpoint the endothelial receptor involved in modulating glioblastoma-derived IL-8-induced permeability. Both CXCR1 and CXCR2 are reported to function as receptors for IL-8, although CXCR2 is predominantly believed to mediate its angiogenic effects [19], [20], [31]. To assess the individual involvement of CXCR1 and CXCR2, RNA interference was used to specifically silence each receptor in hCMEC/D3 (Fig. 6A–B). Interestingly, while knockdown of CXCR1 had no effect on U87-CM-induced permeability (Fig. 6C), silencing of CXCR2 significantly impaired hCMEC/D3 permeability to FITC-dextran (Fig. 6D). Altogether, our data converge on the idea of a glioma secreted-IL-8/endothelial CXCR2 dialogue in hCMEC/D3 permeability.

### Endothelial CXCR2 Conveys Glioblastoma-driven Effects

**Figure 7:**
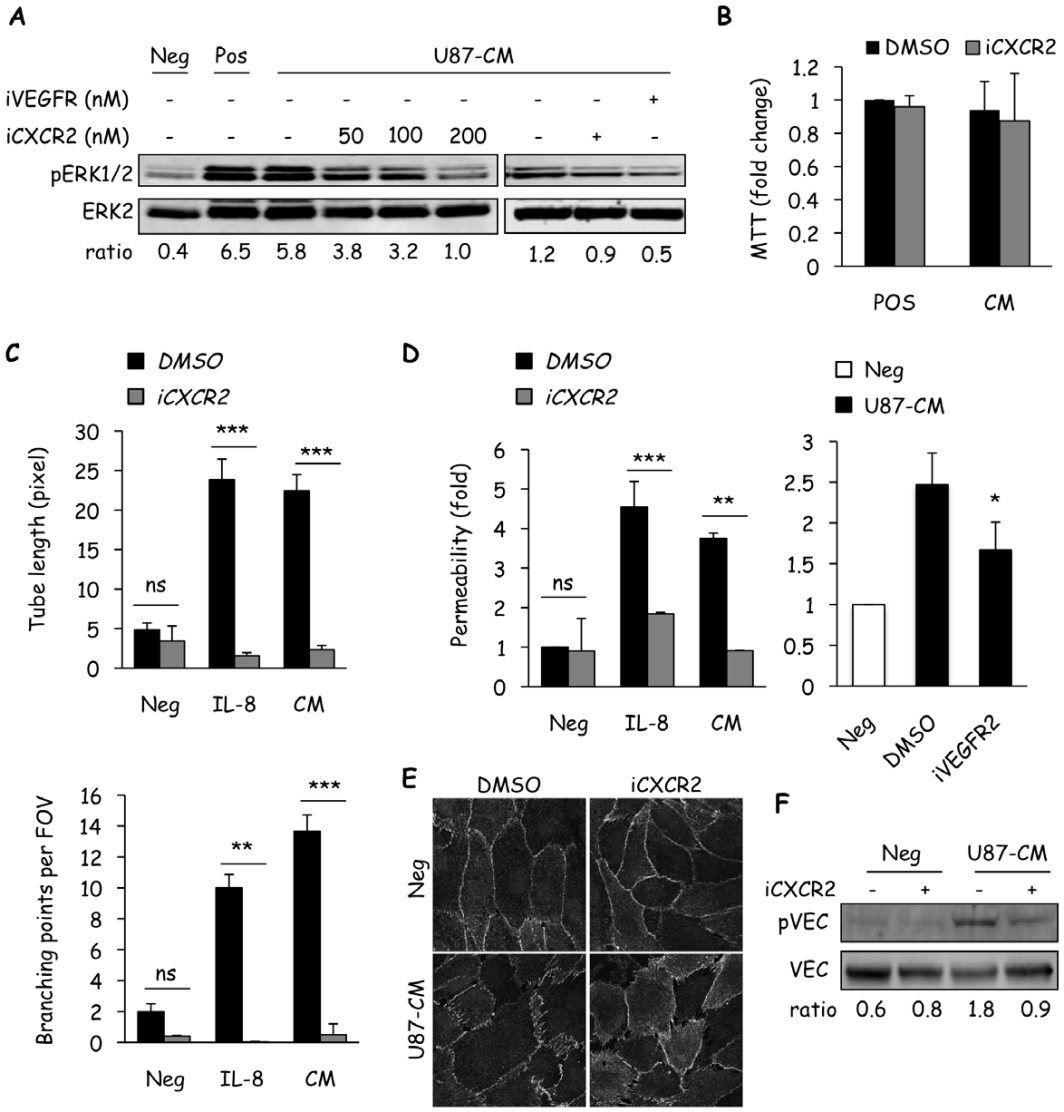
Endothelial CXCR2 conveys glioblastoma cell-driven effects. A) Human cerebral microvascular endothelial cells (hCMEC/D3) were pre-incubated for 45 min with SB225002 (iCXCR2, 200 nM unless indicated) or Ki8751 (iVEGFR2, 10 nM) prior to incubation with U87-CM for 5 min and subsequent determination of ERK activation (pERK). B) The effect of U87-CM, with or without iCXCR2, on the proliferation of hCMEC/D3 was tested by MTT. C–D) The effects of iCXCR2 on U87-CM- or recombinant IL-8-induced tubulogenesis (C) and permeability (D) were investigated. Alternatively, iVEGFR2 was also used in permeability assay. E–F) VE-cadherin (VEC) localization and phosphorylation was examined by confocal (E) and western-blot (F), respectively, after treatment with U87-CM in the presence of iCXCR2 or vehicle (DMSO). For western-blots, ratios between pERK1/2 and ERK2 or pVEC and VEC are indicated below scans. Scale bar: 10 µm. One out of three independent experiments is shown. T test on 3 independent experiments: **p<0.01; ***p<0.001.

To further confirm the requirement of CXCR2 for IL-8/U87-CM-induced effects on hCMEC/D3 brain endothelial cells, a pharmacological inhibitor of CXCR2, SB225002 (iCXCR2) was used. At the molecular level, iCXCR2 dose-dependently decreased U87-CM induced activation of ERK in hCMEC/D3 (Fig. 7A). To further test the potency of iCXCR2 on U87-CM-induced activation of ERK, it was compared with an inhibitor of VEGFR2 signaling (iVEGFR2). Notably, the level of inhibition achieved with iCXCR2 rivaled that of iVEGFR2 (Fig. 7A). We also tested the effects of U87-CM on hCMEC/D3 proliferation in the presence or absence of iCXCR2. Although U87-CM could sustain endothelial cell expansion, the addition of iCXCR2 had no significant effect (Fig. 7B). This suggests that U87-CM is able to substitute for the regular hCMEC/D3 media (Pos), independently of CXCR2 activity. Nevertheless, pharmacological blockade of CXCR2 hampers IL-8- and U87-CM-mediated tubulogenesis *in vitro* (Fig. 7C). Importantly, inhibition of endothelial CXCR2 signaling with SB225002 reduced both recombinant IL-8 (50 ng/ml) and U87-CM-induced permeability of hCMEC/D3 (Fig. 7D), while iVEGFR2 slightly weakened permeability increase (Fig. 7D). Therefore these data suggest that permeability induced through CXCR2 appears to be almost as potent as VEGF pathway-mediated vascular permeability. Similarly, the effect of iCXCR2 on VE-cadherin localization was examined. U87-CM-induced alteration of the VE-cadherin profile in hCMEC/D3 was partially rescued by the presence of iCXCR2 (Fig. 7E) when compared with control. Strikingly, the CXCR2 inhibitor SB225002 strongly abrogated U87-CM-provoked VE-cadherin phosphorylation. Together, these data support the hypothesis that U87-secreted IL-8 is pro-angiogenic and pro-permeability in brain microvascular endothelial cells. Moreover, the IL-8 endothelial receptor CXCR2 is necessary for the phenotype to be executed.

## Discussion

The unique tumor milieu has been characterized for its role as a source of pro-tumorigenic and pro-angiogenic signals [1]. In the present study, to recapitulate some features of the glioblastoma microenvironment *in vitro*, conditioned medium (CM) from the U87 glioblastoma cell line was generated and its effects on the properties of human cerebral microvascular endothelial cells (hCMEC/D3) were studied. We found that U87-CM promoted hCMEC/D3 permeability in correlation with increased phosphorylation and destabilization of VE-cadherin junctions. This also coincided with increased ERK signaling and *in vitro* tubulogenesis suggesting that U87-CM harbors factors, which impart a pro-permeability/pro-angiogenic phenotype on hCMEC/D3. Thus, we analyzed the U87-CM and identified the chemokine interleukin-8 (IL-8, also known as CXCL8) as the most elevated factor in the glioblastoma secretome. Moreover, we demonstrated that interfering with IL-8 function by several complementary approaches, namely siRNA-mediated silencing of IL-8 or its receptor CXCR2, IL-8 blocking antibody, or pharmacological inhibition of endothelial CXCR2, reduced the effects of U87-CM on hCMEC/D3. Therefore, our duet culture model provides evidence that glioblastoma-produced IL-8 elicits a pro-permeability and pro-angiogenic response through its endothelial receptor CXCR2.

At the molecular level, IL-8 activates multiple intracellular signaling pathways downstream of two cell surface G-protein coupled receptors, CXCR1 and CXCR2 [32]. Interestingly, the receptors for IL-8 were more highly expressed in hCMEC/D3 than U87, suggesting that in addition to autocrine signaling [33], U87-secreted IL-8 might elicit effects on endothelial cells *via* CXCR1 and CXCR2. However, siRNA-mediated knockdown of CXCR1 failed to inhibit U87-CM-induced permeability in hCMEC/D3. This suggests that CXCR1 is not significantly involved in modulating the pro-permeability IL-8 signal. In contrast, silencing of CXCR2 impaired the ability of U87-CM to activate ERK, blocked phosphorylation of VE-cadherin and impeded the induction of permeability. Furthermore, the pharmacological inhibitor SB225002 (iCXCR2), which competes with IL-8 for binding to CXCR2, and displays greater than 150-fold selectivity over CXCR1 receptors, abolished the effects of U87-CM on hCMEC/D3. This is in line with previous reports that CXCR2 is chiefly responsible for transmission of IL-8 pro-angiogenic function [17], [34] and correlates with the anti-angiogenic properties of blocking CXCR2 signaling in damaged retina [19]. Indeed, SB225002 treatment not only restricts neovessel formation, but also abrogates edema, fibrogenesis and inflammatory cell recruitment, arguing for a role of IL-8/CXCR2 signaling in vascular normalization [19]. Of note, blocking IL-8 function with siRNA less effectively abrogated the effects of U87-CM. Indeed, a twenty-fold pre-dilution of the CM from IL-8 siRNA-transfected U87 was required in order for the inhibitory effects on hCMEC/D3 to be observed. While this could be inherent to the experimental design, it is interesting to note that the IL-8 family member, Gro-α, which also signals through CXCR2, was also detected in U87-CM by angiogenesis antibody array. Gro-α has been previously reported to exert pro-angiogenic activity [35], thus it might also contribute to the angiogenic phenotype imparted on hCMEC/D3 by the U87 secretome. This may explain why depletion of IL-8 by siRNA in U87-CM did not decrease activation of ERK as dramatically as might be expected, given the level of IL-8 knockdown achieved. This could be attributed as well to VEGF, as blocking VEGFR2 significantly reduced U87-CM-induced ERK phosphorylation. In addition to IL-8 and Gro-α, MCP-1 (also known as CCL2) was also elevated in the U87-CM. MCP-1 has been reported to drive tumor angiogenesis [28]–[30]. Moreover, as its name suggests, MCP-1 recruits monocytes, which might, in turn, release a plethora of cytokines in the tumor microenvironment, exacerbating both angiogenesis and inflammation [36]. However, MCP-1 has also been reported to mediate angiogenesis independently of its pro-inflammatory effect [29]. With respect to permeability, MCP-1 could potentate *in vivo* leakage [37]. In this way, MCP-1 may also contribute to the phenotype elicited by U87-CM on hCMEC/D3.

One of the most striking outcomes observed from stimulating hCMEC/D3 with U87-CM was increased permeability. This phenotype was prevented by either reduction of secreted IL-8 levels or blockade of endothelial CXCR2, suggesting that IL-8 is central in modulating this process. Disturbances in endothelial permeability are frequently observed in cancer where it facilitates angiogenesis, tumor-associated inflammation and metastasis, and correlates with increased interstitial fluid pressure and ensuing edema [7]. Indeed, tumor-associated macrophages have been reported to induce IL-8 secretion by glioblastoma [38]. This intimate relationship between cancer inflammation and tumor angiogenesis suggests that a positive feedback loop may exist in glioblastoma. In this scenario, macrophages might stimulate glioblastoma cells to produce IL-8, which results in increased endothelial permeability. This in turn could lead to the increased flux of inflammatory mediators, which further exacerbate inflammation and endothelial instability in the glioblastoma microenvironment. At the cellular level, elevated permeability is characterized by the redistribution of specialized junction proteins. Indeed, U87-CM induced the spatial re-arrangement of the adherens junction protein, VE-cadherin, and increased its phosphorylation. Importantly, this redistribution of VE-cadherin could be blocked by either specific gene silencing or pharmacological inhibition of the IL-8 receptor, CXCR2, or silencing of IL-8 in U87. Thus, these data support a role for IL-8 in the dynamic remodeling of endothelial junctions during angiogenesis. In addition, we demonstrated that the tumor source of IL-8 can also result in VE-cadherin phosphorylation and monolayer remodeling, and corroborates previous findings that damaged retina uses IL-8/CXCR2 to favor neovascularization [19].

Retrospective analyses of Rembrandt datasets also highlighted that IL-8 is the likely key regulated protein in the IL-8/CXCR2 couplet, as CXCR2 expression levels remained uniform across several brain tumor and non-tumor types, and did not influence the probability of patient survival, while elevated IL-8 levels correlated with reduced likelihood of patient survival. In line with this notion, increased IL-8 signaling confers chemotherapeutic resistance in cancer cells [33]. More recently, it has been suggested that targeting of IL-8 signaling may have important implications for sensitizing tumors to chemotherapeutic and biological agents [15]. Although it does not directly examine chemotherapeutic resistance, analysis of Rembrandt array data strongly suggests that elevated IL-8 contributes to a more aggressive phenotype in brain tumors. Therefore, inhibiting the effects of IL-8 signaling may prove to be a beneficial adjuvant to conventional treatments of glioblastoma. Of note, the CXCR2 inhibitor, SB225002, significantly impaired IL-8/CXCR2-mediated effects on hCMEC/D3. Indeed, we previously demonstrated that the CXCR2 inhibitor, SB225002, inhibits choroidal neovascularization, in association with decreased leakage of fluorescein in mice [19]. Moreover, small molecule antagonists of CXCR2 have previously been used to inhibit the proliferation, survival and angiogenesis of human melanoma tumors grown in mice [39]. Thus, we speculate that SB225002 could be successfully used in a mouse model of glioblastoma.

We also used the Rembrandt library to pool existing gene array data on the expression levels of VEGF, Ang-1 and FGF-2. It is well documented that VEGF is highly expressed in glioblastoma [2]. Indeed, we observed this when using the Rembrandt database (Fig. 3B). Of note, VEGF was not highly detected in the U87-CM using the angiogenesis antibody array. Blockade of VEGF activity with the VEGFR2 inhibitor, Ki8751, however, impaired U87-CM-induced permeability and ERK1/2 activation. This suggests that despite low detectable levels, VEGF still elicits a potent effect, consistent with the clinical benefits of using anti-VEGF therapies, such as Bevazicumab [40]. On the other hand, activation of the IL-8 receptor, CXCR2, might result in co-activation of VEGFR2, even in the absence of VEGF [20]. Thus, inhibition of IL-8 signaling might indirectly impair VEGF signaling. Although we have not specifically examined VEGFR2 activation in response to IL-8 in our model, it is interesting to speculate that IL-8 might function as a master controller to activate both IL-8/CXCR2- and VEGFR-mediated permeability and angiogenesis, and *vice versa*. Of note, hypoxia-mediated activation of IL-8 induces angiogenesis independently of the major oxygen sensor, HIF. This contrasts with VEGF, which is activated by hypoxia in a HIF-dependent manner [41]. Thus, IL-8-induced angiogenesis may act as a compensatory mechanism when HIF is absent [42]. This is important, as many anti-cancer therapies are now focused on the targeted inhibition of HIF and thereby VEGF-mediated angiogenesis [43]–[45]. In the clinic, it is now well described that many brain tumor patients receiving anti-VEGF therapies, such as Bevacizumab, eventually become refractory to treatment and develop more aggressive tumors [46]. Moreover, IL-8 has been reported to underlie patient resistance to the anti-angiogenic agent Sunitinib, which inhibits multiple receptor tyrosine kinases, including VEGFR2 [47]. It is thus possible that tumor cells up-regulate IL-8 to compensate for the reduced VEGF activity, which in turn exploits both the angiogenic CXCR2 pathway, as well as indirectly transactivating the angiogenic VEGFR2 pathway. In this scenario, inhibition of IL-8 signaling could potentially minimize treatment failure or loss of potency. In addition, blocking of the IL-8 receptor CXCR2 would also reduce the effects of other pro-angiogenic chemokines, which signal through this receptor, including Gro-α.

In conclusion, glioblastoma are aggressive, incurable tumors characterized by extensive vascularization. Progression of this cancer is aided by signals from the microenvironment, which includes a variety of cellular components including endothelial cells. In this study, the value of combining *in vitro* models to unravel the discourse, taking place between different cellular constituents has been demonstrated. Furthermore, the chemokine IL-8 has been identified as a major secreted glioblastoma factor which influences endothelial behavior. IL-8 was demonstrated to drive pro-angiogenic and pro-permeability features in brain endothelial cells *via* its cognate receptor, CXCR2. Importantly, blocking IL-8 function by inhibition of its receptor was remarkably efficient at reducing the effects of glioblastoma cell-derived CM on the angiogenic properties of brain microvascular endothelial cells. These findings may have important consequences for the development of anti-angiogenic therapies in glioblastoma, which to-date have met with limited success.
